# Jurassic zircons from the Southwest Indian Ridge

**DOI:** 10.1038/srep26260

**Published:** 2016-05-17

**Authors:** Hao Cheng, Huaiyang Zhou, Qunhui Yang, Lingmin Zhang, Fuwu Ji, Henry Dick

**Affiliations:** 1 State Key Laboratory of Marine Geology, Tongji University, Shanghai 200092, China; 2 Woods Hole Oceanographic Institution, Woods Hole, MA 02543-1050, USA

## Abstract

The existence of ancient rocks in present mid-ocean ridges have long been observed but received less attention. Here we report the discovery of zircons with both reasonably young ages of about 5 Ma and abnormally old ages of approximate 180 Ma from two evolved gabbroic rocks that were dredged from the Southwest Indian Ridge (SWIR) in the Gallieni fracture zone. U–Pb and Lu–Hf isotope analyses of zircons were made using ion probe and conventional laser abrasion directly in petrographic thin sections. Young zircons and their host oxide gabbro have positive Hf isotope compositions (ε_Hf _= +15.7–+12.4), suggesting a highly depleted mantle beneath the SWIR. The spread ε_Hf_ values (from−2.3 to−4.5) of abnormally old zircons, together with the unradiogenic Nd-Hf isotope of the host quartz diorite, appears to suggest an ancient juvenile magmatism along the rifting margin of the southern Gondwana prior to the opening of the Indian Ocean. A convincing explanation for the origin of the unusually old zircons is yet to surface, however, an update of the theory of plate tectonics would be expected with continuing discovery of ancient rocks in the mid-oceanic ridges and abyssal ocean basins.

According to the theory of plate tectonics, ocean crust should not contain rocks older than adjacent oceanic plates in mid-ocean ridges. The discovery of unusually old rocks and minerals in the vicinity of present-day mid-ocean ridges^1^,^2^,^3^, which is far away from the continental margins, requires an alternative mechanism to explain how the ancient rocks have stayed at the present site without being carried away by the spreading oceanic floor. The occurrences of ancient rocks or minerals that were confirmed by absolute dating were sparse and confined in the Mid-Atlantic Ridge so far^3^,^4^. More convincing observations of ancient rocks in mid-ocean ridges of the Atlantic Ocean and other oceans are highly needed before probing their significance for the formation and evolution of the oceans. Recently, several studies, from the Southwest Indian Ridge (SWIR), used ion microprobe U–Pb geochronology of zircon to date the crystallization of igneous rocks intruded into the lower crust in these areas, providing considerable new insight into the time scales of magmatism at mid-ocean ridges^5^,^6^,^7^,^8^. These absolute dating results, however, restricted in a single segment of Atlantis Bank in the SWIR. This paper reports *in-situ* U–Pb age determinations for zircon in petrographic thin sections of a gabbro and a diorite dredged in the Gallieni Fracture Zone at 53°E (Fig. 1). The discovery of Jurassic zircons from the diorite, which has extreme Nd and Hf isotopic ratios consistent with a continental crustal component, suggests preservation of relic continental fragments in the Southwest Indian Ridge.

## Sample and Results

**Figure 1:**
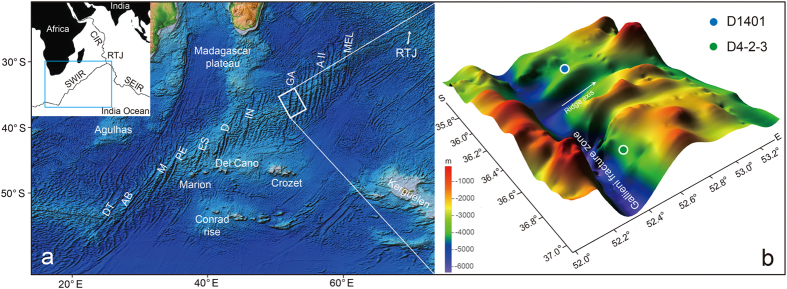
Bathymetric map of the Southwest Indian Ridge floor, showing the locations of samples reported here. (**a**) The base map was prepared using Generic Mapping Tools (GMT)^44^, using ETOPO1 bathymetric data^45^. (**b**) Oblique view of the 52–53°E segment (looking SSE) from *GeoMapApp* (www.geomapapp.org) and sample locations.

The SWIR separates the African and Antarctic plates and is classified as a highly oblique ultraslow-spreading ridge^9^, extending for nearly 8,000 km between the Bouvet and the Indian Ocean Triple Junction (Fig. 1). The rocks studied here are from the 53°E amagmatic segment between the Gallieni fracture zone (FZ) (52^o^20′E) and the Gazelle FZ (53^o^30′E), which is a typical amagmatic segment, and formed at effective full spreading rates <12 km/Myr^9^. This section of the SWIR formed by northeastward propagation of the Indian Ocean Triple Junction between oceanic lithosphere created at the Central Indian and Southeast Indian Ridges^10^. The 53^o^E segment is floored by predominantly mantle peridotites with scarce crustal rocks^11^. This segment of the SWIR has been the focus of several recent dredging expeditions, including the Scripps Institution of Oceanography Indomed Expedition Leg 8, the RV *Marion Dufresne* Cruise 107 and the RV *Dayang Yihao* cruise 21^11^.

The samples examined here were collected during the RV *Dayang Yihao* Cruise 21 in 2010. Sample D4-2-3 (dredge D4-2) is from the transform wall at the end of an east–west ridge at approximately 36^o^40′S south of the spreading centre. Samples D1401 (dredge D1401) were collected on the northern wall of the central rift valley at approximately 35^o^50′S. Sample D4-2-3 is a quartz diorite intruded by plagioclase and quartz-bearing felsic vein (Fig. S1). Mineralogy of the host diorite comprises plagioclase, amphibole, minor pyroxene, quartz, ilmenite and apatite (Table S1, Fig. S1). The felsic vein cross-cuts the diorite and has graphic textures characterized by fine-grained intergrowths of plagioclase and quartz. Sample D1401 is an oxide gabbro, consisting of plagioclase, pyroxene, ilmenite and apatite. Full sample designations and detailed descriptions can be found in Zhou and Dick^11^.

**Figure 2:**
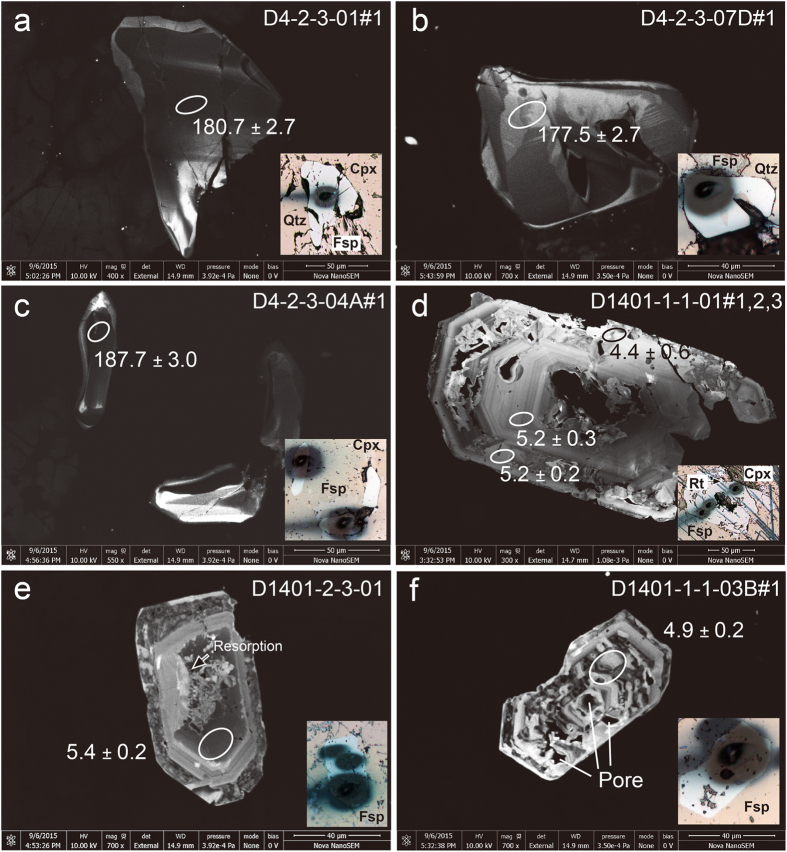
Representative CL images of zircon in petrographic thin sections. Inset photomicrographs showing the location of ion probe pits.

In thin sections, zircon occurs both in the diorite/gabbro and in the felsic vein, locating at the borders between mineral grains or being included in plagioclase (Fig. 2). The analyzed zircons included *in-situ* zircon crystals in thin sections and fragments extracted from whole rocks using the conventional techniques, including crushing, sieving, heavy liquid and hand picking. Individual zircons were mounted in epoxy, polished, and imaged in reflected light, backscattered light, and cathodoluminescence (CL). Additional data and figures are shown in the Supplementary material.

**Figure 3:**
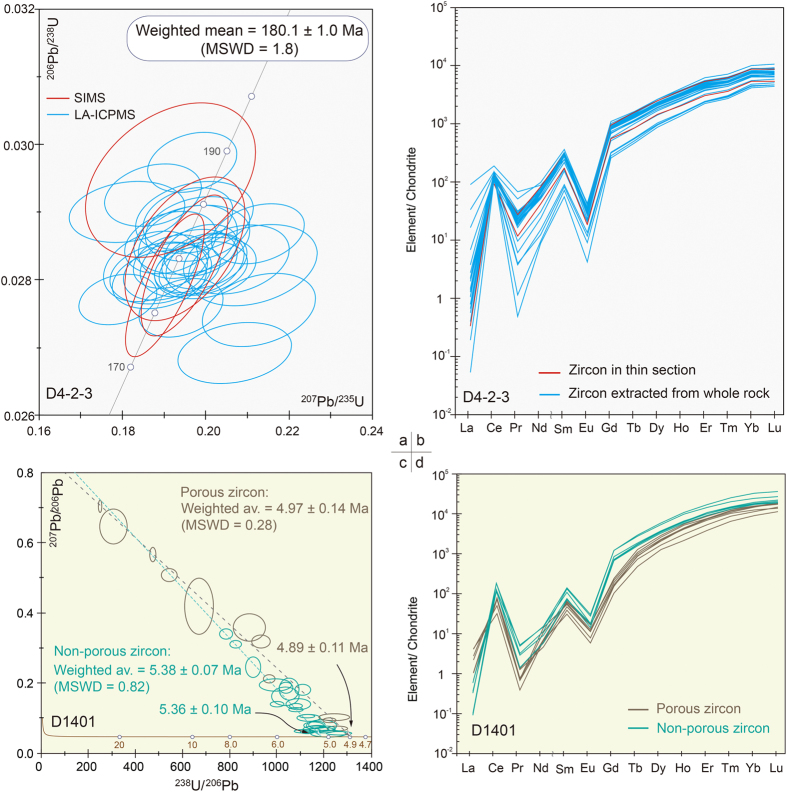
^207^Pb-corrected/^230^Th-disequilibria corrected ages for sample D4-2-3 (**a**–**b**) and D1401 (**c**–**d**).

Zircons from sample D4-2-3 occur as subhedral to anhedral crystals, have unzoned or sector-zoned CL responses (Fig. 2). They show heavy-REE enrichment, positive Ce anomalies and negative Eu anomalies in normalized REE patterns (Fig. 3), high contents of Th (331–3157 ppm) and U (265–3967 ppm), and high Th/U ratios (av. 1.18; Table S2). These observations are characteristic of magmatic zircons^12^. The igneous origin of the zircons, together with the lack of evidence of deformation, recrystallization or metamorphic overgrowths, suggests that the U–Pb zircon ages reflect the timing of igneous crystallization of the sample. Zircon crystallization temperatures range from 963–851 °C with an average value of 903 ± 9 °C using the uncorrected Ti-in-zircon thermometry^13^ (α_(SiO2)_ = 1). Thirty-two analyses on 32 zircon grains are reported in Table 1 and presented in Fig. 3. Analyses yield concordant U–Pb ages, with apparent ^206^Pb/^238^U ages from 188.9 to 175.2 Ma with a weighted mean age of 180.1 ± 1.0 Ma (MSWD = 1.8), which reflects the timing of igneous crystallization of the samples.

Zircons from sample D1401 are mostly euhedral, have sector and fine-scale oscillatory and/or convoluted zoning patterns. All grains are overprinted by secondary textures characterized by cloudy porous features in CL. In contrast to normal magmatic grain (Fig. 2e), zonation within most zircons is often perturbed, convoluted, or chaotic (Fig. 2d,f; Figs S3 and S4). In some instances, perturbed zonation and micro-porosity coincide with specific growth zones within euhedral, otherwise oscillatory zoned zircons, implying alteration of preexisting magmatic grains. Magmatic resorption textures characterized by areas of embayed grain boundaries and secondary luminescent band was observed in some zircons (Fig. 2e), indicating development of younger magmatic overgrowths. Qualitative electron microprobe (EDS) analysis of porous domains displays micron-scale P-, Fe-, Ca- and Cl-rich inclusions, indicating fluid inclusions or salt residues within the pores. The primitive oscillatory zoned, the secondary luminescent and the porous domains define a three stages growth of zircon. The secondary overgrowth is not constrained in this study due to insufficient width for ion probe analysis.

Titanium values largely overlap between the oscillatory zoned and the porous domains (5.4–8.1 ppm vs 5.9–10.9 ppm), which yield Ti-in-zircon temperatures from 748–689 °C with an average value of 711 ± 11 °C for oscillatory domains and 729 ± 21 °C for porous domains, both of which are significantly lower than that of D4-2-3 zircons. The oscillatory zoned and porous domains show similar HREE enrichment relative to the LREEs as well as positive Ce and negative Eu anomalies (Fig. 3), similar to D4-2-3 zircons. Average (Sm/La)_N_ for porous zircon domains is 35 (vs 628 in oscillatory domains), reflecting La enrichment in porous domains. These features, along with the porous and chaotic CL zoning are indicative of interaction with aqueous fluids^14^, which is further evidenced by the higher average (Yb/Gd)_N_ ratio of 78 for the porous domains than the average of 24 for oscillatory domains, resembling that of typical magmatic zircons (~23)^15^,^16^.

U–Pb isotopic analyses were performed on both oscillatory zoned and porous domains. Thirty-six spot analyses on 30 zircon grains were robust and clustered to give well-constrained error-weighted mean ^206^Pb/^238^U ages between 4.3 Ma and 5.8 Ma, showing two distinct age populations between oscillatory zoned and porous domains (Table 2; Fig. 3). A weighted average age of 5.38 ± 0.07 Ma (MSWD = 0.82) and a lower intercept age of 5.36 ± 0.10 are obtained for the oscillatory domains (n = 25), and a weighted average age of 4.97 ± 0.14 Ma (MSWD = 0.28) and a lower intercept age of 4.89 ± 0.11 are defined by 11 analyses on the porous domains. Since the features that characterize porous domains are generally consistent with growth or alteration during aqueous fluid-assisted processes, the weighted-average age of 5.38 ± 0.07 Ma for the oscillatory domains, that are of magmatic in origin, is taken as the best estimate for the timing of igneous crystallization. The weighted mean age of 4.97 ± 0.14 Ma for the porous zircons analyzed is interpreted to reflect crystallization of the vein that intrudes the gabbro.

Hafnium isotope concentrations were collected for 40 zircon grains from sample D4-2-3 and 12 zircons for sample D1401 (Table S3). Initial ^176^Hf/^177^Hf, denoted ε_Hf(*t*)_, is the Hf-isotopic signature of the magma at the time of zircon crystallization and hence reflect that the final derivation of the host rocks from the assumed depleted mantle. The zircon grains from D4-2-3 yielded a restricted spread of initial ε_Hf(*t*)_ values of −2.3 to −4.5 (*t* = 180 Ma), with an average value of −3.2 ± 0.2 (1σ). In contrast, zircons from D1401 have highly positive ε_Hf(*t*)_ values of +15.7 to +12.4 (av. +14.4 ± 0.7 1σ; *t* = 5.4 Ma), plotting at the depleted end of the mantle array^17^ and suggesting a juvenile rock or magma origin from mantle with a moderate- to long-term depletion of Hf relative to Lu.

Nd and Hf isotope compositions of mineral separates and the whole rock of are reported in Table S3. Initial isotopic compositions were calculated at 180 Ma for sample D4-2-3 and 5 Ma for sample D1401. The amphibole from D4-2-3 has negative ε_Nd(*t*)_ (−2.4) and ε_Hf(*t*)_ (−1.9) values, resembling the global Nd–Hf isotope variation of ocean island basalt within the Terrestrial Array^17^, implying that the sample was derived from an enriched mantle source and/or due to crustal contamination or mixing with metasomatized lithospheric mantle. The Nd and Hf isotopic compositions of the whole rock are distinct from those of amphibole. The slightly increased negative ε_Hf(*t*)_ value of the whole rock (−2.6) can be explained by the “zircon effect”^18^ where unradiogenic Hf is locked in zircons and preferentially retained the Hf component even during intense overprinting. The elevated ε_Nd_ value of the whole rock (−1.3) likely reflects the effect of seawater alteration and leaching on Sm–Nd isotope system of ancient submarine rocks. Interestingly, a two-point isochron age of 189 ± 9 Ma, which is coincide with the *c.* 180 Ma zircon U–Pb age within error, is defined by whole rock and clinopyroxene. Sample D1401 has positive ε_Nd_ (+6.8) and ε_Hf_ (+13.3) values, resembling those of the mafic/ultramafic rocks from the 9–25^o^E Southwest Indian Ridge^19^.

## Discussion

Zircons recovered from oceanic diorite and gabbro exposed on the northern wall of the central rift valley, SWIR, typically display oscillatory and sector zoning consistent with igneous crystallization from mafic magmas. There is little magnetic data for the Gallieni Fracture Zone, so directly comparing zircon U–Pb ages with the magnetic ages is difficult. As a first-order approximation, a half-spreading rate of 4.1 ± 0.4 km/Myr is defined by the zircon age (5.38 Ma for sample D1401) versus distance from the ridge axis (~20 km; an along-axis uncertainty of 2 km is estimated based on the valley bathymetry patterns), much lower than the predicted 5.5 km/Myr on the Antarctic and African flanks of SWIR^14^. Anomalously older zircon ages than the predicted magnetic ages for a given portion of crust were reported in several recent studies on Atlantis Bank^6^,^7^, which were commonly interpreted to reflect assimilation of pre-existing gabbroic rocks from the mantle lithosphere^6^. Following this interpretation, the age range of our anomalously old samples (1.3 to 1.7 Myr) would reflect crystallization over depth of ~7 to 9 km below seafloor in the axial valley by assuming a constant spreading rate of 5.5 km/Myr. This depth would be reduced significantly when considering the geometry of the fault^15^,^16^. Alternatively, the anomalously old zircon may reflect sample spacing due to the uncertainty in location by dredging. This interpretation appears to gain support from the observation that the anomalously old zircon relative to the magnetic age, was limited to surface rocks^6^,^7^ and are not found in drill-hole samples in Atlantis Bank^8^. A similar discrepancy between surface and drill-hole sample occurs at Atlantis Massif in the Mid-Atlantic Ridge, where the surface samples yield an anomalously old age, whereas drill-hole samples did not^15^,^16^, supports the latter interpretation. The most likely explanation, however, is that spreading is asymmetric, roughly 10 cm/yr to the south and 4 cm/yr to the north. Similar asymmetries are common along the Southwest Indian Ridge, both by examining isochrons and the EMAG2 magnetic anomaly map^20^ in the *GeoMapApp* database. Such asymmetric north- south spreading has been confirmed at Atlantis Bank by paleomagnetics^21^,^22^,^23^ and zircon age dating^5^,^6^,^8^ where the ridge spreads at ~1 cm/yr to the south, and 4 cm/yr to the north. This is the first time that zircon dating has confirmed asymmetric spreading away from the transform, as in the other cases it is the spreading direction parallel to the transform where this is documented. More distributed surface sampling combined with drilling will clarify the age discrepancy and provide a more accurate estimate of crustal inheritance.

The relatively young ages for the porous zircons may reflect solid-state recrystallization^12^,^24^,^25^ and/or be related to hydrothermal fluid flow associated with intrusion of felsic veins in this area, suggesting that the porous textures developed soon after crystallization of normal magmatic zircons. The initial ^176^Hf/^177^Hf compositions of the normal magmatic grains overlap those of the porous domains, suggesting that fluids catalyzing the formation of the porous zircon domains were derived essentially *in situ* from the magma that crystallized the zircon originally. The sample has a roughly similar range of Hf-isotope ratios, with the local ridge peridotites along the SWIR^19^,^26^, but are more radiogenic than almost all basalts analyzed thus far (except for one basalt from Discovery FZ)^19^.

The SWIR formed with the Mesozoic breakup of Gondwanaland. Zircons from sample D4-2-3, ~60 km south of the axial valley of the SWIR and immediately to the east of the Gallieni Transform fault, have an unusually old U–Pb age of 180.1 ± 1.0 Ma, which is comparable to the initial timing of the opening of the Indian Ocean. Are these rock/zircons erratic or *in-situ*? The age similarity between this ancient sample and the Karoo large igneous province magmatism (179–183 Ma)^27^, which is associated with the breakup of the Gondwanaland at *c.* 180 Ma, apparently suggest an erratic origin, such as ice-rafting and tsunami-depositing, for the ancient rocks. However, the sample location is far away from the continental margins, and it is impossible to sustain the drop-stone theory unless a reasonable explanation is given for the dropping of rocks exclusively on the Gallieni FZ. The sample is thus considered *in-situ* or only slightly moved in origin (see below for further discussion).

Are these rock/zircons relics or formed *in-situ* along the spreading axis? If the rocks/zircons were formed during the generation of the magma, their age would be expected to be about ten million years at most. We thus consider two contrasting possibilities for the origin and the route of the old rock/zircons: 1) An intact Gondwana fragment on the sea-floor ever since formation; 2) A partially altered relic that once resided in shallow regions of the upper mantle, and was entrained and transported to the vicinity beneath the axis. Zircon, residing in shallow regions of the upper mantle on the order of a hundred million years, would significantly lose its radiogenic lead via diffusion^28^, protracted residence of the fragments in the upper mantle is thus highly unlikely.

The ^176^Hf/^177^Hf ratio of 0.282630 ± 0.7 (2SD) for the whole rock of sample D4-2-3 is higher than those of the zircons (0.282609–0.282547). The zircon initial ε_Hf_ values show a variation from −4.5 to −2.3, which is distinct from those of the SWIR peridotites and basalts (+13.9–+6.4)^19^. The unradiogenic Nd isotope (ε_Nd(*t*)_ = −2.4), together with the wide range of initial ε_Hf_ values of zircons, may suggest crustal contamination or interaction between asthenosphere-derived melts and the metasomatized lithospheric mantle in the generation of magmas. The analogous geochronology and isotopic signatures between the ancient diorite and the Karoo magmatic rocks^27^,^29^, imply that the diorite may be correlated with the Jurassic magmatic rocks that emplaced prior to the breakup of southern Gondwana and the opening of the SW Indian Ocean. The rock may represent the relicts onto the margin of the southern Gondwana during the rifting of Africa and Antarctica, possibly a mixture of juvenile and recycled crust in a continental magmatic arc^30^.

Unusually old ages have also been reported from the Mid-Atlantic Ridge^3^,^4^. Several possible mechanisms were introduced to explain the origin of these rocks, mostly of continental affinity, such as ceased ocean-floor^31^, non-spreading blocks^1^, oscillatory spreading^4^, and non-drifting slices^3^. However, all the explanations are difficult to verify, and no convincing data for these hypothetic mechanisms have been given so far. Finding an age of 180 Ma in zircons in a quartz diorite on the SW Indian Ridge is likely to raise considerable doubt as to an ocean ridge origin of the sample. At first glance, it would seem most probable that the sample is a glacial erratic deposited on the seafloor on crust of some ~5 Ma age. Several factors make it possible to reconsider this hypothesis.

First is the condition of the rock, and state of alteration, which are what would be expected for a highly evolved plutonic rock in this particular tectonic setting. Quartz diorites do occur as late differentiates in oceanic gabbro suites, and crosscutting abyssal peridotites. They are not common, but they exist. Highly evolved gabbros and their differentiates are most commonly found as late shallow intrusives into gabbros and peridotites on transform walls where this sample came from, while primitive gabbros are more commonly found near segment centers^32^. Petrographic examination shows mafic minerals are largely replaced by green amphibole and chlorite, while plagioclase appears albitized (Fig. S1). This suggests an upper greenschist facies assemblage that is typical of late high-level intrusive rocks at ocean ridges due to hydrothermal circulation into the crust and shallow mantle near transforms. Moreover, the rock itself shows no signs of glacial scour or rounding which are common in ice-rafted dropstones deposited on the seafloor. So unlike many dropstones investigated by H. Dick over his years’ survey and sampling along the SW India Ridge, the sample is appropriate to its geologic context.

Next is the age of the sample itself. One hundred and eighty million years coincides with the Karoo volcanic event and the initiation of continental breakup. Most of the borderlands to the SW Indian Ridge along the coastline of South Africa are Archean cratonic rocks. However, on the eastern side of Africa and Madagascar lies rocks of the Neoproterozoic Pan-African Orogenic Belt, with their conjugate in East Antarctica. The Pan African Orogenic Belt is an accreted terrain, assembled during the closure of the Mozambique Ocean during the assembly of Gondwana from old island arc terrains and fragments of reworked Archean cratonic rocks. The Karoo volcanic event was widespread, and its remnants are found in South America, Antarctica, Africa and Australia, and would have intruded the Pan African Orogenic Belt during Gondwanan breakup.

There is substantial evidence, based on the geochemistry of the peridotites from the Dragon Bone spreading segment, immediately north of that of the dredged diorite, that the present mantle source of the SW Indian Ridge there represents such old arc-mantle wedge mantle drawn up between the plates along the ridge^30^. The Gallieni FZ nucleated at the margin of a bathymetric plateau lying to the north flanking the eastern side of the Madagascar Plateau. Thus it represents a fracture zone that likely formed by the breakup of an old Gondwanan continental fragment of the Pan-African Orogenic Belt only about 40 to 50 million years ago and is a relatively new and quite shallow ocean basin. Thus, it is reasonable that old Neoproterozoic arc-mantle wedge material, cross intruded by diorite during the initial phase of breakup of Gondwana, was drawn up between the diverging plates and emplaced into the wall of the Gallieni Transform.

Yet to be considered in the discussion is that the quartz diorite is undeformed. Thus, it was not emplaced into its present position in the solid-state, rafted in with delaminated mantle lithosphere. Instead it was intruded into the shallow mantle beneath the rift valley floor, before being unroofed and emplaced onto the transform wall. Mantle peridotites dredged from the Dragon Bone amagmatic segment have universally undergone such deformation, and any intrusive within those rocks would have been deformed with them. However, this was not the normal thermal environment of an ocean ridge segment. First, this amagmatic segment exposes an enormous region of mantle peridotite where the sample was taken. Thus there was little volcanism at the time of emplacement of the diorite and enclosing mantle rocks to the seafloor. This can be ascribed to either very refractory mantle source material or to an extraordinarily cool upper mantle due to ultraslow spreading and the cooling effect of the adjacent 110 km offset Gallieni FZ on the upwelling mantle at the ridge transform intersection. Given the already amagmatic state of spreading, this transform edge effect^33^, would almost totally suppress mantle melting. In such a situation, a quartz-diorite vein, with its very low melting point (~800–1,000 °C) compared to basalt (~1,500–1,250 °C), could be remobilized as the mantle upwelled, while the enclosing peridotite was largely unaffected, to intrude at higher level, carrying its original Gondwanan zircons with it.

This scenario is a reasonable explanation for the emplacement of the quartz diorite, its intrusion into the shallow mantle beneath the rift valley floor, why it is not deformed, but preserves the ancient zircons. It is circumstantial, rather than definitive evidence that permits the possibility that the 180 Ma zircons may have been emplaced *in-situ* – or not.

### Analyses methods and data reduction

Major-element compositions of minerals were analyzed by electron probe microanalysis with a JEOL JXA-8230 Superprobe system at Tongji University. The analyses were performed using an accelerating voltage of 15 kV and beam current of 10 nA, using a 5 μm diameter beam. Natural and synthetic mineral standards were employed for all minerals. JEOL software using ZAF corrections was employed. Representative major-element compositions of feldspar, amphibole, epidote and chlorite are given in Supplemental Table S1.

The U–Pb isotopes for zircons in thin sections were obtained with a Cameca ims1280 microprobe housed at the Institute of Geology and Geophysics, Chinese Academy of Sciences in Beijing. The Pb/U calibration was performed relative to the reference zircon Plešovice, which was analyzed repeatedly throughout each session. Operating conditions and analytical protocols are essentially the same as described by Li *et al*.^34^. A ~20  μm beam was used. U–Pb data for zircon fragments, extracted from rocks using standard crushing, heavy liquid and magnetic separation techniques, and zircon trace element analyses, were obtained by LA–ICP-MS at the State Key Laboratory of Geological Processes and Mineral Resources, China University of Geosciences, using a pulsed 193 nm ArF Excimer laser coupled to an Agilent 7500 ICPMS with a spot size of 32 μm. Zircon standard 91500 was used to normalize fractionation during analysis. External calibration was performed relative to GJ-1 and NIST 610 combined with internal standardization of Si. Off-line selection and integration of background and analytic signals, time-drift correction and quantitative calibration were performed followed that of Liu *et al*.^35^.

A correction for common Pb was made using the ^207^Pb method^36^, and an age appropriate model Pb composition^37^. An additional correction has been made to adjust for initial ^238^U–^230^Th disequilibrium using the equations of Schärer^38^. Zircon commonly hosts ample U relative to Th, which will produce a deficit of radiogenic ^206^Pb due to an initial ^230^Th deficit, and yield a ^206^Pb/^238^U age too young. To correct for these disequilibria for the young sample D1401, the ratio of the mineral/melt partition coefficient for Th and U for zircon (*f* = [Th/U]_zircon_/[Th/U]_magma_) is estimated by dividing the individual spot analyses with the Th/U ratio of bulk rock analyses for rocks collected in this area (Th/U = 2.6 ± 0.5 (95% confidence); data are compiled from *PetDB* (http://www.earthchem.org/petdb). Both ^206^Pb/^238^U and ^207^Pb/^206^Pb ratios are adjusted to account for Th-disequilibria, with these new ratios used to calculate a ^207^Pb corrected ^206^Pb/^238^U age for each analysis of sample D1401.

Zircon Hf isotope analyses were conducted in the the state Key Laboratory of Geological Processes and Mineral Resources, China University of Geosciences in Wuhan, using a Finnigan Neptune plus multi-collector ICP-MS and with a Geolas 2005 excimer ArF laser laser ablation system. All data were acquired on zircon in single spot ablation mode at a spot size of 44 μm. We applied the directly obtained β_Yb_ value from the zircon sample itself in real-time. The ^179^Hf/^177^Hf and ^173^Yb/^171^Yb ratios were used to calculate the mass bias of Hf (β_Hf_) and Yb (β_Yb_), which were normalised to ^179^Hf/^177^Hf = 0.7325 and ^173^Yb/^171^Yb = 1.132685^39^ using an exponential correction for mass bias. Interference of ^176^Yb on ^176^Hf was corrected by measuring the interference-free ^173^Yb isotope and using ^176^Yb/^173^Yb = 0.79639^39^ to calculate ^176^Yb/^177^Hf. Similarly, the relatively minor interference of ^176^Lu on ^176^Hf was corrected by measuring the intensity of the interference-free ^175^Lu isotope and using the recommended ^176^Lu/^175^Lu = 0.02656^40^ to calculate ^176^Lu/^177^Hf. We used the mass bias of Yb (β_Yb_) to calculate the mass fractionation of Lu because of their similar physicochemical properties. The lack of correlation between ^176^Hf/^177^Hf and ^176^Yb/^177^Hf (Fig. S6) is an indicator that the corrections imposed to the ^176^Hf/^177^Hf ratio result in accurate and precise data. Off-line selection and integration of analytic signals, and mass bias calibrations were performed following those of Liu *et al*.^35^.

The Lu–Hf and Sm–Nd isotope analyses for the whole rock and mineral separates were conducted on a ThermoElectron Neptune™ multi-collector (MC–) ICP-MS in the GeoAnalytical Laboratory at Washington State University. About 200 mg of clean, alteration free amphibole, pyroxene and plagioclase were hand-picked under a binocular microscope. Sample dissolution and chemical separations are described by Cheng *et al*.^41^,^42^, and the full data set is presented in Supplementary Tables. Epsilon Hf and Nd values were calculated using ^176^Hf/^177^Hf = 0.282785, ^176^Lu/^177^Hf = 0.0336, ^143^Nd/^144^Nd = 0.512630 and ^147^Sm/^144^Nd = 0.1960 for CHUR^43^.

## Additional Information

**How to cite this article**: Cheng, H. *et al*. Jurassic zircons from the Southwest Indian Ridge. *Sci. Rep.* **6**, 26260; doi: 10.1038/srep26260 (2016).

## Supplementary Material

Supplementary Information

## Figures and Tables

**Table 1:** SIMS and LA–ICPMS zircon U–Pb isotopic data for sample D4-2-3.

*Sample*/	Element (ppm)		Th/U	**Isotopic ratios**						**Ages(Ma)**	
**spot #**	**U**	**Th**		^**206**^**Pb/**^**204**^**Pb**	**1**σ **(%)**	^**207**^**Pb/**^**235**^**U**	**1**σ **(%)**	^**206**^**Pb/**^**238**^**U**	**1**σ **(%)**	**1**σ	
*SIMS*											
01#1	920	1168	1.27	1745	1.5	0.19496	3.0	0.0284	1.5	180.8	2.7
04A#1	759	403	0.53	934	1.6	0.19157	4.4	0.0295	1.6	187.7	3.0
07B#1	1398	1205	0.86	2990	0.8	0.19493	2.2	0.0282	1.5	179.2	2.7
07D#1	331	265	0.80	28742	1.2	0.19019	2.0	0.0279	1.6	177.5	2.7
***LA-ICPMS***				^207^Pb/^206^Pb							
#1	1305	1552	0.86	0.05616	4.4	0.20831	4.4	0.0269	1.1	171.2	1.9
#2	1371	1569	1.15	0.04871	4.2	0.18779	4.1	0.0282	1.2	179.2	2.1
#3	2083	2306	1.23	0.04719	3.6	0.17972	3.5	0.0277	0.9	176.2	1.6
#4	1033	1187	1.87	0.05486	5.0	0.21281	4.9	0.0282	1.4	179.4	2.5
#5	2735	1921	1.49	0.05069	2.9	0.20002	3.0	0.0284	0.9	180.7	1.6
#6	3068	3307	1.72	0.04933	3.2	0.19278	3.1	0.0283	0.9	179.7	1.6
#7	1411	1612	0.48	0.04538	5.2	0.18195	5.1	0.0290	1.0	184.3	1.9
#8	1319	1573	1.47	0.04932	4.5	0.19028	4.3	0.0281	1.1	178.6	1.9
#9	1468	1694	1.90	0.05148	3.8	0.20016	3.8	0.0282	0.9	179.0	1.6
#10	1347	1664	1.99	0.05052	3.9	0.19713	4.0	0.0282	1.2	179.6	2.1
#11	2060	1517	0.50	0.05141	3.4	0.20104	3.4	0.0283	1.0	179.7	1.7
#12	2061	2430	1.79	0.04916	3.5	0.19152	3.5	0.0281	1.0	178.8	1.7
#13	2339	2508	0.39	0.05137	3.1	0.20100	3.2	0.0282	1.0	179.5	1.8
#14	1438	1697	0.24	0.05382	3.4	0.21414	3.5	0.0287	1.0	182.3	1.8
#15	965	1176	1.41	0.05186	4.2	0.20120	4.1	0.0282	1.1	179.1	1.9
#16	3157	3967	1.51	0.05066	2.5	0.19724	2.5	0.0281	0.8	179.0	1.4
#17	1118	1458	1.90	0.05681	4.3	0.21436	4.3	0.0276	1.3	175.2	2.2
#18	1570	1935	0.76	0.04947	4.1	0.18982	4.0	0.0280	1.0	178.0	1.8
#19	1440	1401	0.81	0.04689	4.1	0.18826	4.1	0.0293	1.0	185.8	1.9
#20	1506	1722	1.10	0.05134	3.6	0.19526	3.5	0.0276	1.0	175.4	1.8
#21	1642	1689	1.85	0.04809	3.6	0.19787	3.7	0.0297	1.0	188.9	1.8
#22	883	1015	1.53	0.05071	5.0	0.19793	4.8	0.0286	1.2	181.7	2.1
#23	1489	1854	0.96	0.04902	3.9	0.19267	3.8	0.0286	1.1	181.5	1.9
#24	1072	800	1.16	0.05036	4.8	0.19845	4.8	0.0289	1.2	183.8	2.2
#25	1412	1647	1.00	0.05081	4.4	0.19927	4.3	0.0287	1.1	182.7	2.0
#26	1585	1332	0.81	0.04937	3.7	0.19695	3.7	0.0289	1.0	183.7	1.9
#27	1155	1060	1.29	0.05028	4.0	0.19689	4.1	0.0286	1.3	181.5	2.2
#28	1039	1213	1.27	0.05351	4.1	0.20882	4.1	0.0283	1.2	179.9	2.1

**Table 2:** SIMS U–Pb isotopes for zircons from sample D1401.

*Sample*/	[U]	[Th]	Th/U	^206^Pb^a^	^238^U^b^	±σ	^207^Pb b	±σ	corrected^c^	±σ
**spot #**	ppm	ppm		^204^Pb	^206^Pb	%	^206^Pb	%	age (Ma)	
***D1401***										
3-1-07#1	417	333	0.80	42.6	782.2	2.9	0.3408	3.4	5.2	0.4
3-1-06#1	398	286	0.72	42.8	823.5	2.4	0.3126	2.8	5.2	0.4
3-1-05#2	149	105	0.71	73.5	1189	4.4	0.0795	17.3	5.3	0.2
3-1-05#1	364	274	0.75	55.6	965.3	2.1	0.2135	4.6	5.3	0.2
3-1-04#1	256	126	0.49	315	1257	2.8	0.0721	7.7	5.0	0.1
3-1-03#2	119	32	0.27	150	1163	3.8	0.0702	10.8	5.5	0.2
3-1-03#1	425	342	0.81	91.6	1078	2.1	0.1360	4.1	5.4	0.1
3-1-02A#2	280	170	0.61	109	998.4	2.4	0.1411	5.0	5.8	0.2
3-1-02A#1	448	723	1.61	225	1127	2.2	0.0618	5.7	5.6	0.1
3-1-01#01	347	323	0.93	220	1264	3.5	0.0577	7.0	5.1	0.2
1-1-07#1	565	647	1.15	256	1173	2.4	0.0768	4.8	5.3	0.1
1-1-06#1	388	122	0.32	35.9	666.4	7.5	0.4201	15.5	5.2	1.1
1-1-05#5	458	471	1.03	112	1167	2.5	0.0823	9.2	5.3	0.1
1-1-05#4	250	118	0.47	52.4	898.0	2.9	0.2474	9.7	5.4	0.4
1-1-05#3	172	108	0.63	71.5	1051	3.2	0.1838	13.6	5.1	0.3
1-1-05#2	154	118	0.77	56.4	1134	3.5	0.1019	7.9	5.4	0.2
1-1-05#1	309	49	0.16	59.8	931.7	3.5	0.3203	4.7	4.6	0.4
1-1-04B#1	263	342	1.30	248	1111	2.9	0.0640	7.3	5.7	0.2
1-1-04A#1	372	209	0.56	118	1109	2.4	0.1828	7.0	4.9	0.2
1-1-03C#4	214	155	0.73	139	1155	3.1	0.0803	10.1	5.4	0.2
1-1-03C#3	160	141	0.88	55.6	992.5	5.7	0.1964	7.2	5.3	0.4
1-1-03C#2	198	131	0.66	29.2	540.8	5.2	0.5085	2.9	5.0	1.0
1-1-03C#1	93	56	0.61	39.6	1033	4.6	0.1632	13.3	5.4	0.3
1-1-03B#2	177	124	0.70	25.2	470.3	2.1	0.5666	2.9	4.7	1.2
1-1-03B#1	152	119	0.78	86.6	1192	3.8	0.0660	9.6	5.3	0.2
1-1-03A#1	171	117	0.68	21.9	245.9	2.6	0.7047	2.0	4.3	2.9
1-1-02#2	500	263	0.53	110	1242	4.3	0.1036	7.4	4.9	0.2
1-1-01#3	80	34	0.43	31.0	880.8	6.3	0.3600	8.9	4.5	0.6
1-1-01#2	201	151	0.75	133	1217	5.1	0.0575	13.8	5.3	0.3
1-1-01#1	85	34	0.40	60.8	1090	4.0	0.1408	9.2	5.3	0.2
3-1-02B#1	732	306	0.42	168	1223	1.9	0.0956	5.5	5.0	0.1
2-1-01#1	260	94	0.36	19.3	302.8	15.8	0.6464	6.3	5.1	2.5
2-1-03#1	535	775	1.45	107	1079	2.3	0.1248	3.7	5.4	0.2
2-1-06B#1	1209	1274	1.05	239	1128	2.2	0.1069	3.5	5.3	0.1
2-1-08#1	418	256	0.61	232	1210	2.5	0.0715	7.4	5.2	0.1
3-1-09A#1	382	362	0.95	56.7	1037	2.2	0.1886	6.8	5.2	0.2

^a^Measured values.

^b^Uncorrected ratios.

^c^^207^Pb-corrected and ^230^Th-disequilibria corrected ages.
