# Supplementary material for: Jurassic zircons from the Southwest Indian Ridge
Source: Sci Rep. 2016 May 17;6:26260. doi: 10.1038/srep26260 (PMC4869104; doi:10.1038/srep26260)

**Supplementary**

**Title:** Jurassic zircons from the Southwest Indian Ridge

**Authors:** Hao Cheng^*^, Huaiyang Zhou, Qunhui Yang, Lingmin Zhang, Fuwu Ji & Henry Dick

chenghao@tongji.edu.cn | oahgnehc@gmail.com

**Table S1. Representative major element compositions (wt %) of minerals in diorite.**

| SiO_2_ | TiO_2_ | Al_2_O_3_ | Cr_2_O_3_ | FeO | MnO | MgO | CaO | Na_2_O | K_2_O | Total |
| --- | --- | --- | --- | --- | --- | --- | --- | --- | --- | --- |
| *Feldspar* |  |  |  |  |  |  |  |  |  |  |
| 66.83 | 0.00 | 20.00 | 0.03 | 0.07 | 0.00 | 0.02 | 0.88 | 11.21 | 0.09 | 99.13 |
| 66.23 | 0.00 | 19.77 | 0.01 | 0.04 | 0.00 | 0.03 | 0.95 | 10.86 | 0.09 | 97.98 |
| 63.14 | 0.03 | 18.16 | 0.02 | 0.02 | 0.00 | 0.00 | 0.00 | 0.33 | 15.96 | 97.66 |
| 61.14 | 0.00 | 20.09 | 0.02 | 0.07 | 0.01 | 0.00 | 0.00 | 0.30 | 15.36 | 96.98 |
| 62.61 | 0.08 | 17.89 | 0.05 | 0.05 | 0.01 | 0.02 | 0.09 | 0.56 | 15.36 | 96.72 |
| 62.14 | 0.00 | 17.94 | 0.02 | 0.01 | 0.00 | 0.00 | 0.06 | 0.56 | 15.47 | 96.20 |
| 63.54 | 0.03 | 18.73 | 0.06 | 0.00 | 0.03 | 0.00 | 0.03 | 0.63 | 15.48 | 98.53 |
| 66.06 | 0.00 | 19.61 | 0.00 | 0.02 | 0.00 | 0.00 | 0.59 | 10.72 | 0.12 | 97.11 |
| 63.12 | 0.00 | 18.14 | 0.02 | 0.10 | 0.00 | 0.00 | 0.02 | 0.23 | 15.98 | 97.62 |
| 57.31 | 0.00 | 26.29 | 0.02 | 0.11 | 0.00 | 0.03 | 7.83 | 6.67 | 0.19 | 98.43 |
| 58.43 | 0.00 | 25.40 | 0.01 | 0.17 | 0.00 | 0.01 | 6.80 | 7.48 | 0.23 | 98.52 |
| 58.33 | 0.00 | 25.48 | 0.04 | 0.10 | 0.04 | 0.00 | 7.18 | 6.98 | 0.19 | 98.34 |
| 58.10 | 0.02 | 25.50 | 0.02 | 0.12 | 0.00 | 0.00 | 7.39 | 7.31 | 0.25 | 98.70 |
| 57.64 | 0.01 | 25.46 | 0.05 | 0.14 | 0.00 | 0.03 | 7.29 | 6.90 | 0.25 | 97.76 |
| 57.35 | 0.03 | 25.36 | 0.04 | 0.19 | 0.03 | 0.00 | 7.11 | 7.04 | 0.31 | 97.42 |
| 65.32 | 0.00 | 17.56 | 0.02 | 0.05 | 0.00 | 0.02 | 0.01 | 0.51 | 15.40 | 98.88 |
| 63.22 | 0.01 | 18.06 | 0.02 | 0.04 | 0.02 | 0.00 | 0.07 | 0.52 | 15.48 | 97.44 |
| *Amphibole* |  |  |  |  |  |  |  |  |  |  |
| 49.16 | 0.09 | 4.36 | 0.08 | 15.29 | 0.41 | 12.00 | 11.32 | 0.70 | 0.27 | 93.68 |
| 48.47 | 0.00 | 4.43 | 0.06 | 15.41 | 0.43 | 12.21 | 11.46 | 0.74 | 0.27 | 93.46 |
| 48.97 | 0.07 | 6.44 | 0.08 | 17.14 | 0.36 | 11.87 | 11.28 | 1.04 | 0.41 | 97.65 |
| 48.67 | 0.15 | 5.35 | 0.02 | 16.51 | 0.43 | 11.79 | 11.35 | 0.72 | 0.34 | 95.35 |
| 48.53 | 0.22 | 4.22 | 0.10 | 16.96 | 0.38 | 11.32 | 11.34 | 0.65 | 0.31 | 94.02 |
| 49.08 | 0.21 | 4.21 | 0.02 | 16.42 | 0.33 | 12.19 | 11.74 | 0.55 | 0.25 | 95.01 |
| 46.64 | 0.06 | 5.53 | 0.04 | 16.88 | 0.40 | 11.28 | 11.20 | 0.82 | 0.42 | 93.26 |
| 49.66 | 0.19 | 4.70 | 0.00 | 16.78 | 0.35 | 12.07 | 11.46 | 0.65 | 0.36 | 96.23 |
| 47.97 | 0.16 | 5.10 | 0.30 | 16.40 | 0.25 | 11.44 | 11.28 | 0.81 | 0.43 | 94.13 |
| 48.68 | 0.23 | 5.11 | 0.00 | 17.70 | 0.40 | 11.09 | 11.27 | 0.85 | 0.39 | 95.71 |
| 50.32 | 0.08 | 3.15 | 0.01 | 16.08 | 0.43 | 13.08 | 10.86 | 0.67 | 0.21 | 94.90 |
| 49.37 | 0.42 | 3.84 | 0.08 | 15.16 | 0.41 | 13.17 | 11.72 | 0.78 | 0.25 | 95.20 |
| 49.37 | 0.12 | 3.94 | 0.03 | 16.55 | 0.45 | 12.49 | 10.51 | 0.91 | 0.23 | 94.59 |
| 49.06 | 0.07 | 4.14 | 0.02 | 15.94 | 0.33 | 12.27 | 10.75 | 1.02 | 0.27 | 93.87 |
| 49.59 | 0.02 | 4.05 | 0.05 | 16.71 | 0.48 | 12.50 | 10.95 | 0.84 | 0.25 | 95.45 |
| 48.92 | 0.08 | 3.81 | 0.05 | 15.75 | 0.52 | 12.69 | 10.97 | 0.78 | 0.26 | 93.82 |
| 49.06 | 0.42 | 4.15 | 0.04 | 17.27 | 0.49 | 11.88 | 11.00 | 0.92 | 0.28 | 95.51 |
| 49.98 | 0.36 | 5.52 | 0.05 | 16.91 | 0.40 | 11.42 | 11.14 | 0.82 | 0.72 | 97.31 |
| 48.77 | 0.15 | 6.21 | 0.07 | 17.45 | 0.45 | 12.49 | 11.33 | 0.96 | 0.44 | 98.31 |
| 48.21 | 0.06 | 7.24 | 0.13 | 17.23 | 0.32 | 12.03 | 11.05 | 1.16 | 0.43 | 97.87 |
| 48.33 | 0.20 | 4.79 | 0.07 | 16.43 | 0.35 | 11.65 | 11.25 | 0.79 | 0.35 | 94.21 |
| 50.69 | 0.25 | 3.34 | 0.01 | 15.79 | 0.35 | 11.87 | 11.45 | 0.53 | 0.22 | 94.49 |
| 48.59 | 0.29 | 4.83 | 0.07 | 17.07 | 0.44 | 11.53 | 11.21 | 0.70 | 0.43 | 95.16 |
| *Epidote* |  |  |  |  |  |  |  |  |  |  |
| 38.75 | 0.08 | 27.43 | 0.02 | 6.13 | 0.07 | 0.06 | 22.99 | 0.07 | 0.14 | 95.73 |
| 38.04 | 0.08 | 27.17 | 0.00 | 6.02 | 0.08 | 0.09 | 22.77 | 0.07 | 0.14 | 94.46 |
| 37.45 | 0.02 | 26.36 | 0.03 | 7.66 | 0.39 | 0.02 | 22.20 | 0.00 | 0.00 | 94.13 |
| 37.51 | 0.04 | 25.72 | 0.00 | 8.56 | 0.05 | 0.09 | 23.09 | 0.00 | 0.01 | 95.07 |
| 38.93 | 0.00 | 24.97 | 0.04 | 9.29 | 0.10 | 0.06 | 22.72 | 0.05 | 0.01 | 96.17 |
| 37.12 | 0.02 | 22.86 | 0.00 | 12.07 | 0.03 | 0.00 | 22.96 | 0.04 | 0.01 | 95.08 |
| 36.56 | 0.10 | 20.99 | 0.08 | 13.87 | 0.09 | 0.04 | 22.40 | 0.02 | 0.04 | 94.17 |
| 36.96 | 0.23 | 21.91 | 0.01 | 12.89 | 0.11 | 0.05 | 22.36 | 0.04 | 0.00 | 94.55 |
| 36.69 | 0.17 | 19.43 | 0.00 | 15.66 | 0.09 | 0.05 | 22.15 | 0.00 | 0.01 | 94.24 |
| 36.70 | 0.13 | 21.88 | 0.00 | 13.02 | 0.08 | 0.12 | 22.47 | 0.00 | 0.02 | 94.40 |
| 36.65 | 0.17 | 22.71 | 0.00 | 11.76 | 0.17 | 0.25 | 22.48 | 0.04 | 0.01 | 94.23 |
| *Chlorite* |  |  |  |  |  |  |  |  |  |  |
| 26.82 | 0.04 | 17.28 | 0.15 | 24.70 | 15.36 | 0.33 | 0.06 | 0.09 | 0.07 | 84.88 |
| 26.66 | 0.00 | 17.25 | 0.12 | 24.40 | 15.28 | 0.28 | 0.13 | 0.16 | 0.09 | 84.35 |
| 26.40 | 0.04 | 17.02 | 0.03 | 24.86 | 14.96 | 0.25 | 0.01 | 0.05 | 0.02 | 83.64 |
| 26.41 | 0.02 | 17.15 | 0.00 | 25.01 | 14.89 | 0.29 | 0.05 | 0.00 | 0.04 | 83.86 |
| 25.95 | 0.48 | 17.30 | 0.08 | 24.98 | 14.42 | 0.28 | 0.52 | 0.09 | 0.07 | 84.16 |
| 26.56 | 0.10 | 17.81 | 0.08 | 25.66 | 14.82 | 0.29 | 0.09 | 0.07 | 0.14 | 85.61 |
| 26.48 | 0.00 | 16.81 | 0.04 | 25.47 | 14.90 | 0.15 | 0.04 | 0.00 | 0.10 | 83.99 |
| 26.10 | 0.00 | 17.73 | 0.04 | 23.78 | 14.12 | 0.27 | 0.00 | 0.13 | 0.08 | 82.23 |
| 26.25 | 0.02 | 17.80 | 0.01 | 24.51 | 14.60 | 0.28 | 0.07 | 0.07 | 0.09 | 83.71 |
| 26.10 | 0.06 | 17.84 | 0.12 | 25.35 | 14.74 | 0.34 | 0.04 | 0.00 | 0.01 | 84.58 |
| 26.07 | 0.03 | 17.96 | 0.06 | 25.09 | 14.55 | 0.28 | 0.00 | 0.01 | 0.06 | 84.09 |
| 26.59 | 0.05 | 16.96 | 0.03 | 23.88 | 15.37 | 0.29 | 0.04 | 0.02 | 0.03 | 83.24 |
| 25.35 | 0.05 | 17.36 | 0.09 | 24.87 | 14.47 | 0.24 | 0.06 | 0.01 | 0.03 | 82.53 |
| 27.70 | 1.98 | 16.24 | 0.07 | 22.27 | 14.01 | 0.26 | 2.52 | 0.03 | 0.17 | 85.25 |
| 26.72 | 0.03 | 16.92 | 0.00 | 26.50 | 14.28 | 0.29 | 0.00 | 0.02 | 0.04 | 84.80 |
| *Ilmenite* |  |  |  |  |  |  |  |  |  |  |
| 0.05 | 46.00 | 0.03 | 0.19 | 44.48 | 2.65 | 0.07 | 0.00 | 0.00 | 0.00 | 93.48 |
| *Sphene* |  |  |  |  |  |  |  |  |  |  |
| 29.74 | 31.47 | 2.72 | 0.05 | 1.48 | 0.04 | 0.04 | 26.93 | 0.07 | 0.01 | 92.55 |
| 30.58 | 23.08 | 9.08 | 0.06 | 1.91 | 0.00 | 0.07 | 28.63 | 0.06 | 0.01 | 93.48 |

**Table S2. Zircon (sample D4-2-3) REE data obtained by LA-ICPMS.**

| spot | La | Ce | Pr | Nd | Sm | Eu | Gd | Tb | Dy | Ho | Er | Tm | Yb | Lu | Ti |
| --- | --- | --- | --- | --- | --- | --- | --- | --- | --- | --- | --- | --- | --- | --- | --- |
| ***D4-2-3*** | |  |  |  |  |  |  |  |  |  |  |  |  |  |  |
| #1 | 1.72 | 79.0 | 2.13 | 25.8 | 34.8 | 1.85 | 148 | 44.0 | 497 | 163 | 702 | 124 | 1171 | 182 | 54.2 |
| #2 | 0.27 | 85.2 | 2.20 | 32.8 | 49.1 | 1.67 | 183 | 52.0 | 578 | 184 | 787 | 140 | 1283 | 199 | 44.9 |
| #3 | 0.08 | 89.4 | 1.88 | 27.5 | 42.9 | 1.93 | 175 | 49.8 | 565 | 183 | 795 | 144 | 1355 | 211 | 45.4 |
| #4 | 0.32 | 70.9 | 2.85 | 37.8 | 50.5 | 2.41 | 181 | 49.3 | 548 | 176 | 734 | 129 | 1194 | 182 | 65.8 |
| #5 | 0.14 | 89.4 | 0.11 | 4.69 | 11.4 | 0.54 | 60.4 | 19.9 | 253 | 89.0 | 418 | 81.0 | 825 | 136 | 31.9 |
| #6 | 0.10 | 97.8 | 1.60 | 30.3 | 43.5 | 2.08 | 174 | 52.4 | 602 | 200 | 889 | 160 | 1513 | 241 | 56.4 |
| #7 | 0.33 | 80.4 | 2.30 | 30.6 | 44.7 | 1.37 | 182 | 51.5 | 588 | 191 | 829 | 144 | 1321 | 206 | 48.9 |
| #8 | 0.53 | 80.4 | 2.60 | 36.2 | 49.0 | 1.62 | 186 | 55.2 | 616 | 199 | 843 | 147 | 1373 | 211 | 42.5 |
| #9 | 0.35 | 85.7 | 2.09 | 30.6 | 43.1 | 1.39 | 179 | 52.0 | 586 | 188 | 805 | 140 | 1322 | 204 | 79.4 |
| #10 | 0.69 | 76.3 | 2.93 | 37.2 | 48.9 | 2.04 | 212 | 60.8 | 692 | 225 | 962 | 165 | 1532 | 239 | 31.1 |
| #11 | 0.17 | 87.9 | 0.05 | 4.30 | 8.71 | 0.25 | 55.5 | 18.3 | 228 | 81.0 | 380 | 72.0 | 726 | 117 | 47.6 |
| #12 | 0.20 | 95.2 | 2.34 | 33.6 | 46.9 | 2.13 | 195 | 58.0 | 656 | 215 | 916 | 164 | 1523 | 235 | 46.5 |
| #13 | 0.26 | 92.2 | 1.79 | 31.3 | 49.1 | 1.57 | 188 | 53.7 | 620 | 202 | 868 | 155 | 1442 | 223 | 59.4 |
| #14 | 4.18 | 93.4 | 3.14 | 32.9 | 44.8 | 1.90 | 172 | 49.2 | 556 | 179 | 761 | 135 | 1266 | 194 | 51.0 |
| #15 | 0.35 | 74.0 | 2.58 | 36.6 | 48.2 | 2.55 | 189 | 55.8 | 603 | 196 | 823 | 144 | 1339 | 206 | 79.6 |
| #16 | 0.57 | 96.3 | 2.23 | 31.8 | 44.8 | 1.88 | 190 | 58.8 | 698 | 239 | 1057 | 188 | 1742 | 277 | 52.5 |
| #17 | 8.40 | 99.4 | 5.13 | 48.2 | 57.4 | 2.48 | 231 | 63.7 | 706 | 228 | 963 | 167 | 1514 | 236 | 56.5 |
| #18 | 0.37 | 84.0 | 2.46 | 35.8 | 46.4 | 2.12 | 203 | 58.7 | 656 | 219 | 925 | 161 | 1501 | 232 | 65.9 |
| #19 | 0.05 | 78.4 | 0.94 | 14.9 | 26.3 | 1.25 | 108 | 32.8 | 371 | 125 | 550 | 100 | 966 | 153 | 51.8 |
| #20 | 0.27 | 88.9 | 2.21 | 32.2 | 47.9 | 1.69 | 191 | 53.6 | 599 | 190 | 799 | 144 | 1340 | 204 | 54.3 |
| #21 | 0.01 | 82.3 | 0.60 | 11.4 | 24.6 | 1.23 | 123 | 38.4 | 440 | 145 | 639 | 117 | 1122 | 172 | 34.1 |
| #22 | 0.50 | 66.6 | 1.94 | 24.7 | 34.9 | 1.82 | 149 | 42.0 | 467 | 152 | 653 | 115 | 1097 | 168 | 57.3 |
| #23 | 0.08 | 85.0 | 2.72 | 39.9 | 48.5 | 1.95 | 203 | 60.8 | 662 | 218 | 925 | 162 | 1494 | 233 | 54.4 |
| #24 | 0.01 | 60.4 | 1.16 | 20.2 | 27.4 | 1.11 | 119 | 32.9 | 380 | 121 | 526 | 96.0 | 919 | 143 | 46.8 |
| #25 | 0.95 | 91.0 | 2.57 | 36.1 | 48.2 | 1.95 | 193 | 54.2 | 592 | 188 | 795 | 142 | 1318 | 202 | 52.4 |
| #26 | 0.20 | 77.3 | 0.39 | 6.08 | 13.1 | 0.69 | 66.1 | 21.5 | 256 | 88.0 | 404 | 76.0 | 765 | 123 | 48.5 |
| #27 | 0.01 | 69.2 | 0.37 | 7.27 | 14.2 | 0.80 | 69.5 | 22.3 | 271 | 92.0 | 412 | 79.0 | 784 | 124 | 40.6 |
| #28 | 0.37 | 86.8 | 2.01 | 27.5 | 38.2 | 1.87 | 156 | 44.9 | 505 | 164 | 694 | 124 | 1168 | 182 | 40.4 |
| *D1401* | |  |  |  |  |  |  |  |  |  |  |  |  |  |  |
| #1 *^p^* | 0.26 | 45.4 | 0.06 | 3.20 | 9.29 | 0.96 | 46.0 | 40.6 | 676 | 297 | 1290 | 305 | 2551 | 451 | 5.35 |
| #2 *^p^* | 0.26 | 51.2 | 0.07 | 3.38 | 9.90 | 0.97 | 49.0 | 48.2 | 739 | 314 | 1470 | 336 | 2626 | 464 | 6.77 |
| #3 *^p^* | 0.17 | 47.4 | 0.13 | 2.79 | 7.21 | 0.65 | 35.0 | 32.3 | 562 | 236 | 1167 | 262 | 2089 | 352 | 8.12 |
| #4 *^p^* | 0.54 | 47.2 | 0.12 | 2.19 | 5.96 | 0.46 | 31.0 | 33.8 | 526 | 241 | 1218 | 285 | 2466 | 471 | 7.85 |
| #5 *^p^* | 0.69 | 47.6 | 0.06 | 5.12 | 8.72 | 0.75 | 39.0 | 36.4 | 581 | 253 | 1284 | 309 | 2568 | 490 | 6.78 |
| #6 *^p^* | 1.00 | 32.4 | 0.07 | 6.76 | 8.24 | 0.68 | 33.0 | 27.7 | 429 | 173 | 886 | 223 | 1933 | 379 | 8.06 |
| #7 *^p^* | 1.04 | 20.4 | 0.04 | 3.11 | 4.69 | 0.34 | 21.0 | 18.1 | 321 | 124 | 654 | 167 | 1503 | 294 | 6.82 |
| #8 | 0.02 | 114 | 0.30 | 5.33 | 20.0 | 1.56 | 250 | 108 | 1461 | 606 | 2804 | 635 | 5398 | 921 | 7.67 |
| #9 | 0.08 | 113 | 0.46 | 7.18 | 21.4 | 1.73 | 245 | 100 | 1316 | 551 | 2400 | 511 | 4081 | 691 | 9.69 |
| #10 | 0.14 | 78.0 | 0.49 | 6.96 | 16.1 | 1.02 | 171 | 68.9 | 923 | 375 | 1677 | 360 | 2915 | 497 | 10.9 |
| #11 | 0.09 | 69.8 | 0.28 | 4.26 | 11.3 | 0.83 | 136 | 58.0 | 801 | 320 | 1529 | 335 | 2777 | 481 | 9.43 |
| #12 | 0.02 | 78.6 | 0.13 | 2.80 | 10.7 | 1.00 | 147 | 64.3 | 910 | 364 | 1744 | 385 | 3175 | 553 | 10.0 |
| #13 | 0.02 | 75.1 | 0.13 | 2.80 | 10.6 | 1.05 | 143 | 62.3 | 864 | 350 | 1665 | 366 | 3005 | 523 | 5.85 |

Units: ppm. b.d. *^p^*: porous.

**Table S3. LA–ICP-MS Lu–Hf isotope analysis of zircons.**

| *Sample*/ | ^176^Yb/^177^Hf | ^176^Lu/^177^Hf | ^176^Hf^/177^Hf | ±σ | ε_Hf(_*_0_*)*^*^* | ε_Hf(_*_t_*) |
| --- | --- | --- | --- | --- | --- | --- |
| ***D4-2-3*** |  |  |  |  |  | *t = 180 Ma* |
| #1 | 0.020842 | 0.000702 | 0.282593 | 0.000016 | -6.8 | -2.9 |
| #2 | 0.028793 | 0.000947 | 0.282581 | 0.000015 | -7.2 | -3.3 |
| #3 | 0.032526 | 0.001096 | 0.282593 | 0.000013 | -6.8 | -2.9 |
| #4 | 0.015559 | 0.000534 | 0.282579 | 0.000013 | -7.3 | -3.4 |
| #5 | 0.022760 | 0.000774 | 0.282609 | 0.000018 | -6.2 | -2.3 |
| #6 | 0.027504 | 0.000906 | 0.282602 | 0.000013 | -6.5 | -2.6 |
| #7 | 0.029386 | 0.000970 | 0.282607 | 0.000015 | -6.3 | -2.4 |
| #8 | 0.025643 | 0.000863 | 0.282567 | 0.000016 | -7.7 | -3.8 |
| #9 | 0.028221 | 0.000930 | 0.282581 | 0.000016 | -7.2 | -3.3 |
| #10 | 0.022565 | 0.000755 | 0.282589 | 0.000014 | -6.9 | -3.0 |
| #11 | 0.030480 | 0.001004 | 0.282608 | 0.000012 | -6.3 | -2.4 |
| #12 | 0.022637 | 0.000749 | 0.282590 | 0.000012 | -6.9 | -3.0 |
| #13 | 0.015820 | 0.000545 | 0.282575 | 0.000013 | -7.4 | -3.5 |
| #14 | 0.024359 | 0.000800 | 0.282593 | 0.000019 | -6.8 | -2.9 |
| #15 | 0.028060 | 0.000919 | 0.282579 | 0.000015 | -7.3 | -3.4 |
| #16 | 0.027788 | 0.000919 | 0.282582 | 0.000017 | -7.2 | -3.3 |
| #17 | 0.017960 | 0.000605 | 0.282547 | 0.000014 | -8.4 | -4.5 |
| #18 | 0.014576 | 0.000496 | 0.282569 | 0.000016 | -7.6 | -3.7 |
| #19 | 0.025626 | 0.000833 | 0.282604 | 0.000016 | -6.4 | -2.5 |
| #20 | 0.029586 | 0.000958 | 0.282606 | 0.000019 | -6.3 | -2.4 |
| #21 | 0.026933 | 0.000873 | 0.282595 | 0.000017 | -6.7 | -2.8 |
| #22 | 0.030617 | 0.001035 | 0.282571 | 0.000019 | -7.6 | -3.7 |
| #23 | 0.041134 | 0.001349 | 0.282599 | 0.000021 | -6.6 | -2.7 |
| #24 | 0.018516 | 0.000619 | 0.282558 | 0.000017 | -8.0 | -4.1 |
| #25 | 0.022318 | 0.000746 | 0.282588 | 0.000011 | -7.0 | -3.0 |
| #26 | 0.028723 | 0.000941 | 0.282575 | 0.000019 | -7.4 | -3.5 |
| #27 | 0.013482 | 0.000458 | 0.282586 | 0.000013 | -7.0 | -3.1 |
| #28 | 0.025245 | 0.000844 | 0.282587 | 0.000023 | -7.0 | -3.1 |
| #29 | 0.032456 | 0.001059 | 0.282569 | 0.000017 | -7.6 | -3.8 |
| #30 | 0.026519 | 0.000869 | 0.282590 | 0.000017 | -6.9 | -3.0 |
| #31 | 0.012555 | 0.000435 | 0.282585 | 0.000013 | -7.1 | -3.1 |
| #32 | 0.012677 | 0.000432 | 0.282578 | 0.000017 | -7.3 | -3.4 |
| #33 | 0.033723 | 0.001110 | 0.282582 | 0.000021 | -7.2 | -3.3 |
| #34 | 0.023402 | 0.000780 | 0.282569 | 0.000020 | -7.6 | -3.7 |
| #35 | 0.011203 | 0.000386 | 0.282583 | 0.000012 | -7.1 | -3.2 |
| #36 | 0.027634 | 0.000919 | 0.282572 | 0.000016 | -7.5 | -3.6 |
| #37 | 0.022819 | 0.000760 | 0.282582 | 0.000013 | -7.2 | -3.3 |
| #38 | 0.027494 | 0.000896 | 0.282589 | 0.000012 | -6.9 | -3.0 |
| #39 | 0.019472 | 0.000656 | 0.282578 | 0.000014 | -7.3 | -3.4 |
| #40 | 0.010514 | 0.000369 | 0.282565 | 0.000015 | -7.8 | -3.8 |
| ***D1401*** |  |  |  |  |  | *t = 5.4 Ma* |
| 2-3-1-D1 | 0.099781 | 0.003581 | 0.283134 | 0.000104 | +12.4 | +12.5 |
| 2-3-1-D2 | 0.178358 | 0.005749 | 0.283163 | 0.000064 | +13.4 | +13.5 |
| 1A *^p^* | 0.197347 | 0.006742 | 0.283132 | 0.000045 | +12.3 | +12.4 |
| 1B *^p^* | 0.065594 | 0.002201 | 0.283199 | 0.000017 | +14.6 | +14.7 |
| 1C *^p^* | 0.078027 | 0.002707 | 0.283215 | 0.000031 | +15.2 | +15.3 |
| 3A *^p^* | 0.077844 | 0.003062 | 0.283196 | 0.000032 | +14.5 | +14.6 |
| 3B *^p^* | 0.189956 | 0.006250 | 0.283192 | 0.000029 | +14.4 | +14.5 |
| 4 *^p^* | 0.231781 | 0.007416 | 0.283226 | 0.000028 | +15.6 | +15.7 |
| 5 *^p^* | 0.158412 | 0.005259 | 0.283204 | 0.000027 | +14.8 | +14.9 |
| 6 | 0.114039 | 0.003979 | 0.283172 | 0.000036 | +13.7 | +13.8 |
| *^*^* ^176^Hf/^177^Hf_CHUR(0)_ = 0.282875 and ^176^Lu/^177^Hf_CHUR(0)_ = 0.0336 (Bouvier et al., 2008). λ^176^Lu = 1.867x10^-11^ (Scherer | | | | | | |
| et al., 2001; Söderlund et al., 2004). *^p^*: analysis on porous domain. | | | | | | |

**Table S4. Lu–Hf and Sm–Nd isotope data for sample D4-2-3.**

| Sample *^a^* | Lu (ppm) | Hf (ppm) | ^176^Lu/^177^Hf *^b^* | ^176^Hf/^177^Hf *^c^* | Sm (ppm) | Nd (ppm) | ^147^Sm/^144^Nd *^b^* | ^143^Nd/^144^Nd *^c^* |  |
| --- | --- | --- | --- | --- | --- | --- | --- | --- | --- |
| *D4-2-3* |  |  |  |  |  |  |  |  |  |
| Amphibole | 1.22 | 1.35 | 0.128 | 0.283050 ± 5 | 8.61 | 40.5 | 0.1287 | 0.512430 ± 10 |  |
| Feldspar | 0.021 | 0.165 | 0.0184 | 0.280319 ± 15 | 2.61 | 4.70 | 0.3355 | 0.512351 ± 12 |  |
| Relics | 0.182 | 0.279 | 0.0927 | 0.282749 ± 4 | 3.28 | 16.5 | 0.1206 | 0.515412 ± 10 |  |
| whole rock | 0.250 | 3.83 | 0.00928 | 0.282630 ± 7 | 4.39 | 73.5 | 0.0361 | 0.512375 ± 10 |  |
|  |  |  |  |  |  |  |  |  |  |
| *D1401* |  |  |  |  |  |  |  |  |  |
| Pyroxene | 9.99 | 0.213 | 6.69 | 0.298238 ± 6 | 2.02 | 5.87 | 0.2082 | 0.512931 ± 9 |  |
| Feldspar | 0.020 | 0.062 | 0.0490 | 0.281913 ± 34 | 0.17 | 0.55 | 0.1869 | 0.513045 ± 10 |  |
| Relics | 0.111 | 0.365 | 0.0433 | 0.282916 ± 4 | 0.74 | 1.95 | 0.2297 | 0.513025 ± 7 |  |
| whole rock | 1.52 | 4.71 | 0.0517 | 0.283162 ± 6 | 0.98 | 2.62 | 0.2275 | 0.512981 ± 10 |  |
| *^a^* Relics, mixture of remained minerals after picking of amphibole/pyroxene and feldspar. | | | | | | | | | |
| *^b^* Uncertainties for ^176^Lu/^177^Hf and ^147^Sm/^144^Nd for the purpose of regressions and calculations is estimated to be 0.5%. | | | | | | | | | |
| *^c^* Reported errors on the ^176^Hf/^177^Hf and ^143^Nd/^144^Nd are within-run 2σ, standard error, and are given in the 6^th^ decimal place. | | | | | | | | | |

**Figure S1. Photomicrographs of ancient quartz diorite (D4-2-3) from SWIR.**

**
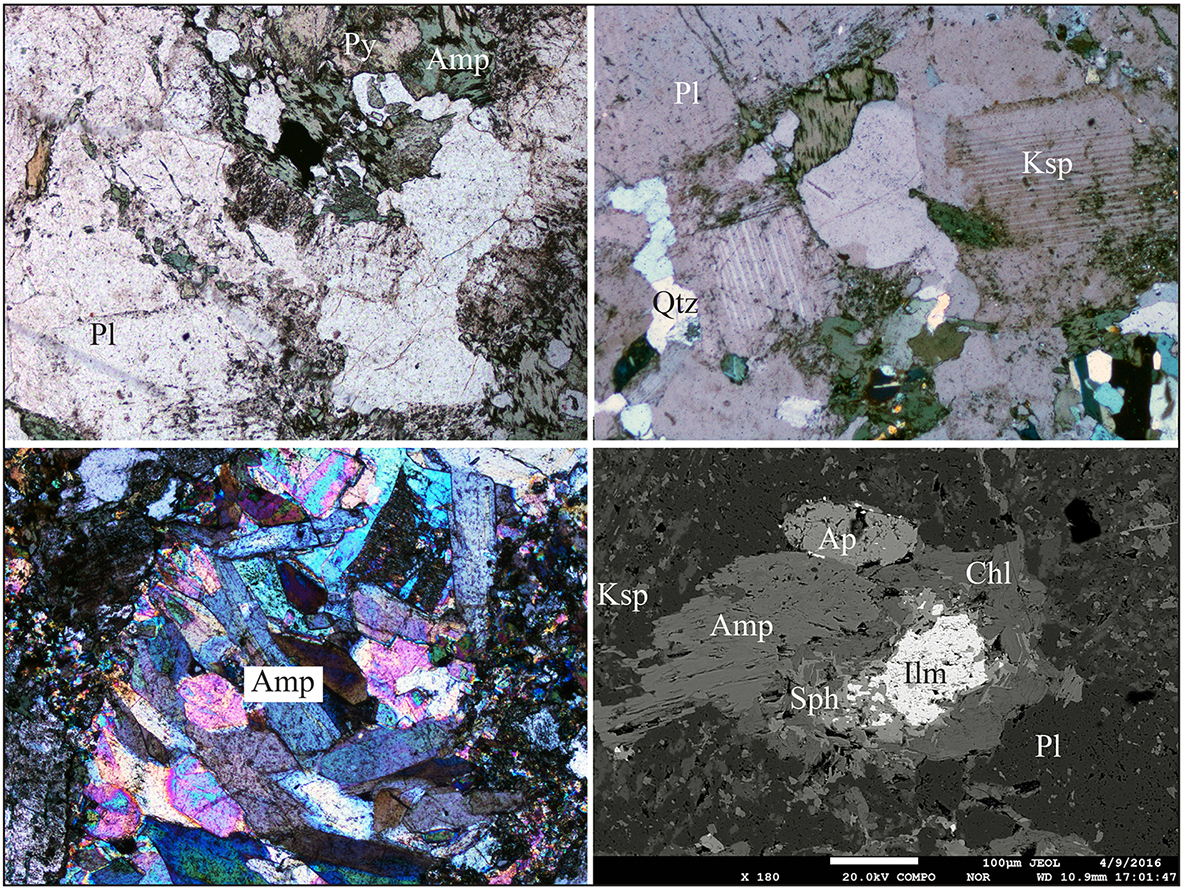
**

**Figure S2. Zircon (D4-2-3-01#1 in Fig. 2) in the thin section.**


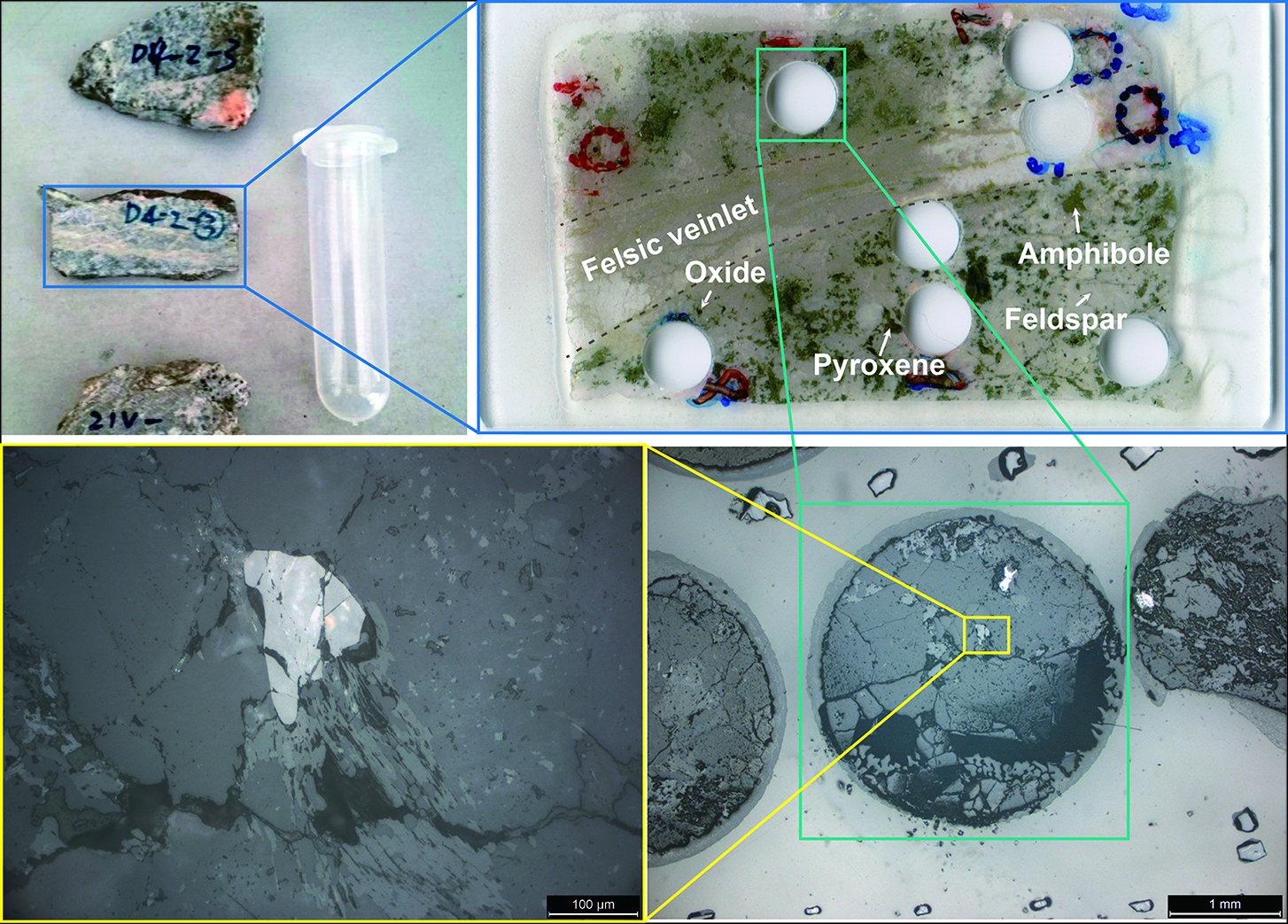


**Figure S3. Representative CL images of zircon from sample D1401 and corresponding apparent ages (Ma).**


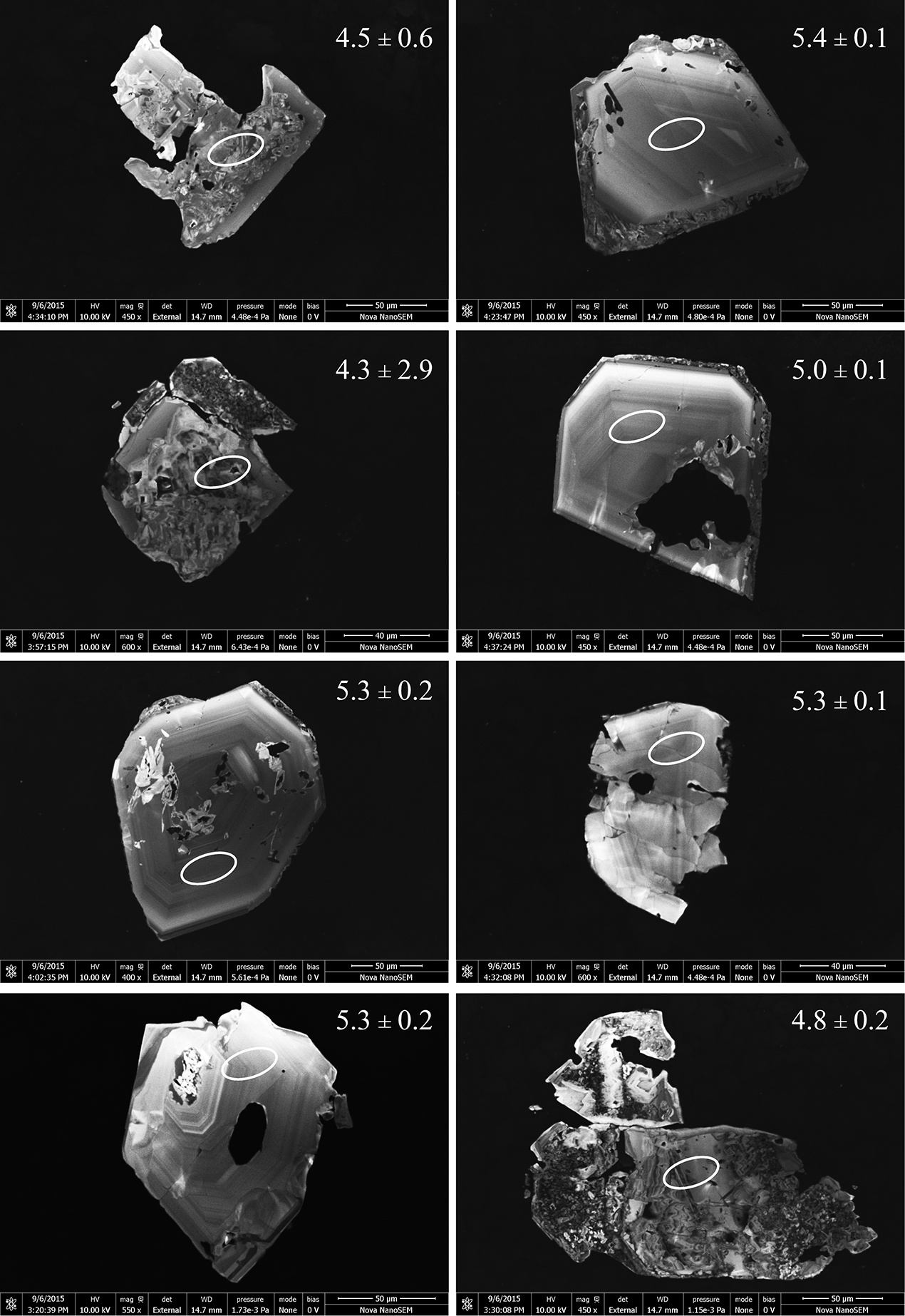


**Figure S4. Representative CL images of zircon from sample D4-2-3 and corresponding apparent ages (Ma).**

**
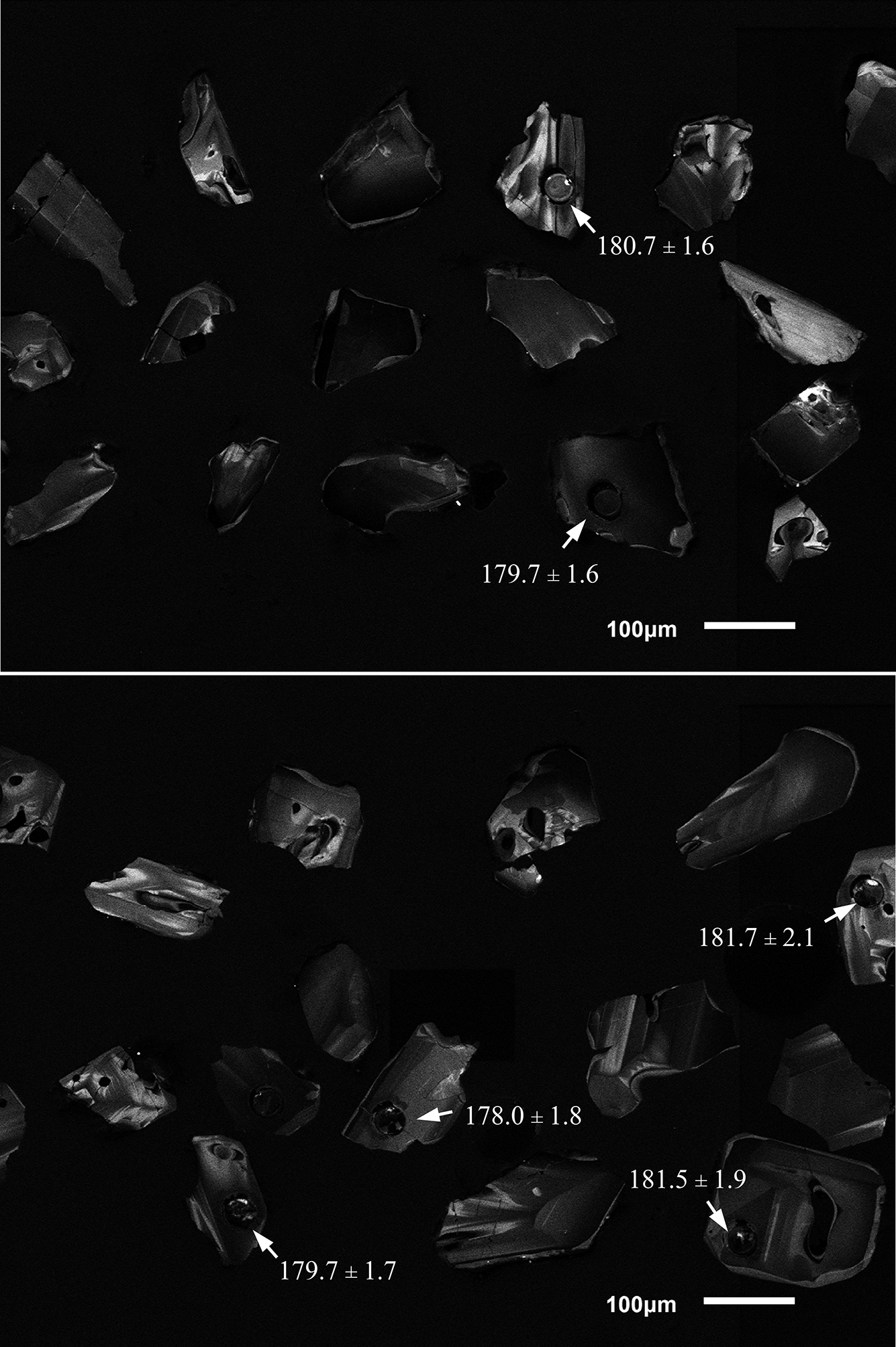
**

**Figure S5. Analytical results for the ^176^Hf/^177^Hf isotope ratios on 91500 by LA−ICP-MS.** **Green line stands for the reference value (Wu et al., 2006).**


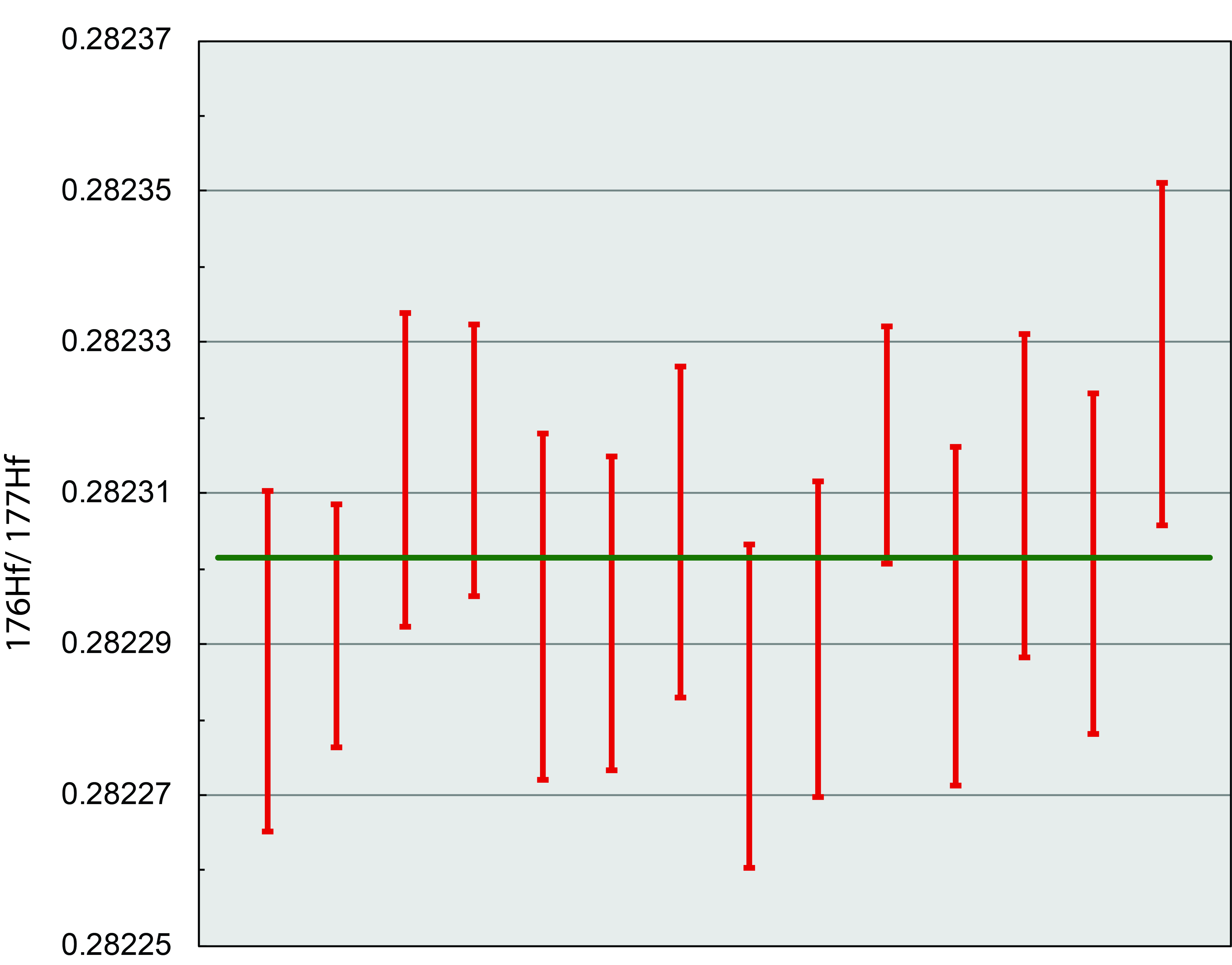


**Figure S6. Plot of ^176^Yb/^177^Hf *vs*. ^176^Hf/^177^Hf.**


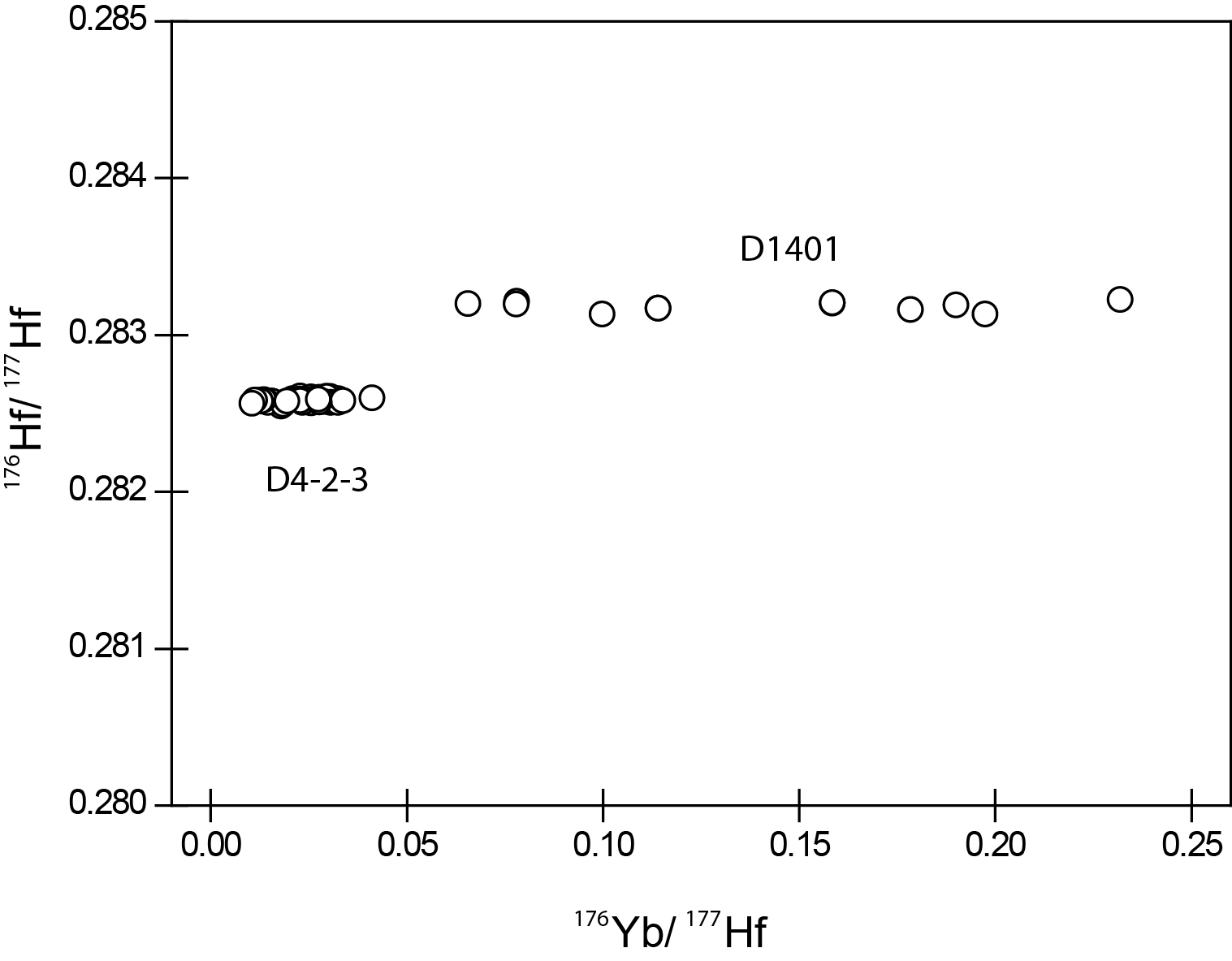

Supplement: Supplementary Information [file srep26260-s1.doc]
